# Enriching limited information on rare diseases from heterogeneous networks for drug repositioning

**DOI:** 10.1186/s12911-021-01664-x

**Published:** 2021-11-16

**Authors:** Hongkui Cao, Liang Zhang, Bo Jin, Shicheng Cheng, Xiaopeng Wei, Chao Che

**Affiliations:** 1 Key Laboratory of Advanced Design and Intelligent Computing, Ministry of Education, Dalian University, Dalian, 116622 China; 2 International Business College, Dongbei University of Finance and Economics, Dalian, 116025 China; 3 School of Innovaton and Entrepreneurship, Dalian University of Technology, Dalian, 116024 China; 4 School of Computer Science and Technology, Dalian University of Technology, Dalian, 116024 China

**Keywords:** Rare diseases, Drug repositioning, Heterogeneous networks, Biased random walk

## Abstract

**Background:**

The historical data of rare disease is very scarce in reality, so how to perform drug repositioning for the rare disease is a great challenge. Most existing methods of drug repositioning for the rare disease usually neglect father–son information, so it is extremely difficult to predict drugs for the rare disease.

**Method:**

In this paper, we focus on father–son information mining for the rare disease. We propose GRU-Cooperation-Attention-Network (GCAN) to predict drugs for the rare disease. We construct two heterogeneous networks for information enhancement, one network contains the father-nodes of the rare disease and the other network contains the son-nodes information. To bridge two heterogeneous networks, we set a mapping to connect them. What’s more, we use the biased random walk mechanism to collect the information smoothly from two heterogeneous networks, and employ a cooperation attention mechanism to enhance repositioning ability of the network.

**Result:**

Comparing with traditional methods, GCAN makes full use of father–son information. The experimental results on real drug data from hospitals show that GCAN outperforms state-of-the-art machine learning methods for drug repositioning.

**Conclusion:**

The performance of GCAN for drug repositioning is mainly limited by the insufficient scale and poor quality of the data. In future research work, we will focus on how to utilize more data such as drug molecule information and protein molecule information for the drug repositioning of the rare disease.

## Background

A disease is defined as a rare disease if it affects less than 200,000 people in the United States [1], or less than 1/2000 of the population in Europe [2]. According to a global report of rare diseases, many people may be affected by one of about 6000 known rare diseases in the world [3]. Therefore, the treatment of rare diseases is very important and significant. But the rare disease lacks the important information including the drug molecule information, the gene information, the protein three-dimensional structure information. Thus, the treatment of rare diseases is difficult to be found, how to complete drug repositioning for rare diseases is a valuable problem.

At present, drug repositioning methods are mainly divided into three types [4]: (1) Structure-based methods [5], (2) Ligands similarity-based methods [6], (3) Machine learning-based methods [7]. Structure-based methods mainly focus on the molecular information of complexes in biology. For example, AutoDock [8], proposed by Morris in 2009, combines long experience freedom and lamarckian genetic algorithm for modeling, which makes full use of the information of protein’s molecular structure to predict the relationship between ligands and protein through genetic algorithm. AutoDock usually requires the detailed molecular information and three-dimensional structure of proteins, however, many existing rare disease-related proteins are not yet known, which limits the development of such methods for drug repositioning. Some methods that based on the similarity of ligands: which use a large number of known protein ligands and then calculate the similarity score of each group of ligands. But ligands similarity-based methods may leaks data information in the processing of predicting results, and the accuracy of the prediction model is far from the actual accuracy. Since this method has irreversible high-risk problems, ligands similarity-based methods are not suitable for rare diseases [9].

Deep learning-based models with higher predictive capacity have also been developed in various drug discovery settings [10–15]. MSCMF, proposed by Zheng in 2013, calculates the similarity of related drugs and genes through matrix factorization operation, but these operations could cause a lot of information lost, which would affect the entire model. Some methods like DTINet [7] proposed by Luo in 2017 and HNM [16] proposed by Wang in 2014, which could greatly improve the accuracy of prediction. But these methods still could not solve the problem of limited information in data. DTINet uses an unsupervised way to learn low-dimensional feature representations of related drugs and genes from Heterogeneous Network(HN) data, and uses an induction matrix [17]. DTINet may not be sufficient to capture the complex hidden features behind HN data. Recent advances in information transfer and aggregation techniques extend Convolutional Neural Networks(CNN) to large-scale graphical data, which significantly improves the predictive performance of models associated with HNs and helps us use deep learning models to discover complex information from HNs. Some of methods mentioned above have good performance in the field of drug repositioning [18, 19], but those methods for drug repositioning [20] usually requires lots of labelled data for training. With the development of Internet medical services in recent years, more and more medical knowledge is stored in the form of HNs. How to fully explore the data in HNs for drug repositioning is very important. The first step to utilize the knowledge in HNs is to use HN embedding method to represent the knowledge.

PtransE is one of the traditional path-level HN embedding methods. Compared with the TransE [21] model, it adds relational reasoning to HN embedding. However, our method pays more attention to the relationship sequence than the entity sequence, which causes the loss of entity information. Many methods only focus on the information of entity or relationship [22–24]. Different from these methods, our method uses DeepWalk [25] in the HN embedding and uses a unified random walk to sample the path in the networks, which can fully mine the path information from HN data. node2vec [26] uses biased random walk to enhance the path’s sampling of HNs. Our method can smoothly control the direction of walking, which could be biased towards depth-first search or breadth-first search. The mechanism that we propose in this paper that is biased samples the father–son relationships is inspired by node2vec, father–son nodes mean some disease nodes that have a hierarchical relationship, the distance between these nodes is within two hops, and they belong to the same type of disease in the biological definition. Many HN embedding methods usually focus on clusters or communities of related nodes without considering the semantics and direction of the relationship, such as structure2vec [27], SSE [28] and JK-Net [29].

HN embedding is already a mature research topic, the Trans-series model is proposed for translational embedding, such as TransE, TransH [30] and TransR [31]. ComplEx [32] enhanced the basic DistMult [33] model through embedding HNs into the complex space. RotatE [34] is a rotation that defines each relationship as a head entity to a tail entity. Some recent studies have shown that HN embedding can also improve the performance of entity alignment models. MtransE [35] can train different HN embedding separately and learns the transition between HN embedding. BootEA [36] is a method of entity alignment based on HN embedding by using a fine algorithm to update the alignment in the iterative process. KDCoE [37] is an HN embedding method that trains entity relationships and semantics together, but it requires additional pre-training multilingual word embedding and description. GCN-Align [38] is a HN embedding method of neighboring neighborhoods based on Graph Convolutional Networks (GCN). But our method does not consider the semantics of relations between entities. In the above methods, the TransE-based model is difficult to obtain the dependence of the long-term relationship of HNs, and it is difficult to disseminate information between different HNs. The GCN-based network does not use the semantic information of the relationship. Recurrent Skipping Networks (RSN) [39] network can alleviate the above problems, but it is difficult to obtain the hierarchical information of the nodes, which leads to insufficient performance of the model in the very sparse HN data.

To solve the problem of limited information, we extract disease data matching rare diseases from open data source and merge it with real data from hospital. The data are transformed into tuples of HNs, so that the relationship between nodes can be found through the path-level information between different nodes [40]. We mainly focus on rare disease nodes with hierarchical relationships and the nodes within two hops, named as father–son nodes. We design a biased random walk mechanism to collect the information of father–son nodes, which is helpful to explore the possibility of treatment of rare diseases with conventional drugs.

The main contributions of our paper lie in three points:

1. We have realized drug repositioning for rare diseases with limited information through public data and the data of Peking Union Medical College Hospital.
2. We use path-level information to predict the nodes in rare disease data from two HNs and enhance the connection between them.
3. We use the Gated Recurrent Unit (GRU) network to strengthen the weights of nodes near the source node and input its output to the attention network for optimization, thus making full use of the limited information.

## Methods

### Problem formulation

In a HN, nodes with hierarchical relationship and distance less than two hops are called father–son nodes. A drug often has a therapeutic effect on the diseases that have a father–son relationship. For example, Gaucher type III is a sub-category of gaucher disease, both imiglucerase and taliglucerase alfa could cure the two diseases. To make full use of the father–son relationship, we use a combination of two HNs to embed data. We set father nodes and son nodes in HN1 and HN2, respectively. The two HNs are connected by a matrix with two columns. One column indicates father nodes of HN1, the other column indicates son nodes of HN2. We use a path-based model with biased random walk mechanism to smoothly sample the path information of related nodes, which can obtain the path information between father nodes and son nodes.

We choose GRU [41] network to model the related path. Since the current output of the GRU network only depends on the output of the previous node and the current input, which could ignore the role of closely related nodes in sequence prediction. On this basis, we add the father node information and the neighboring node information in the current path information to the hidden information through a cooperative mechanism to help the model predict the drug. Experiments prove that our model GCAN has excellent performance in the drug repositioning of rare diseases.

To solve the problem of drug repositioning for rare diseases, we propose a new method named as GRU-Cooperation-Attention-Network (GCAN). We use the biased random walk mechanism to control our model to collect the information of father–son nodes smoothly. What’s more, we present a sampling method to enrich the information of rare disease. GCAN use the cooperation mechanism based on GRU units to make full use of data, we processed the outputs of GRU units by attention mechanism to further improve the computing power of the model.

GCAN is a prediction model based on deep learning, which consists of three parts: (a) biased random walk, (b) cooperation mechanism, (c) attention mechanism. We first use (a) biased random walk to collect information from HN, which is more inclined to collect the information about father–son nodes. Then, (b) cooperation mechanism is employed to enhance the ability of prediction for GRU network. What’s more, to further improve the ability of prediction for model, we use (c) attention mechanism to enhance the model and improve the ability of our model.

### Path-level embedding

The rare disease nodes are usually independent of each other and not connected to a HN. Therefore, we select some related common diseases in the same format to form a certain scale of HN. Finally, we constructed a network with complex path relationships. Due to the scarcity of information on rare diseases, we processed all data in the form of [disease, gene, drug], which is more useful to mine the hidden information. There are two types of relationships between the nodes: the pathogenic relationship between a disease and a gene, the therapeutic relationship between a drug and a disease. However, HNs with only a small amount of data are difficult to train models with higher accuracy. In this case, it is easy to cause prediction errors in drug repositioning. In view of the unity of the relationship in the existing data, we use genes as the connection between diseases and drugs, and enrich the types of relationship through the diversity of genes. At this time, we process the data into a triple format: T = (h, r, t), where h and t represent the disease entity and the drug entity, and r represents the gene connecting the disease and the drug [21]. The traditional methods explore the shift-invariance of head entity for embedding network, tail entity and relation in the vector space. However, the potential information contained in the triple data is too scarce, and the model is difficult to find the favorable information for prediction, so we model a longer relationship chain information to obtain hidden information of long-distance related nodes. We use biased random walk to collect the information from nodes, it would be more like to collect father–son nodes, which is useful for model to get important information. Thus, we can get a sequence $(X_1^t, X_2^r, X_3^t, \ldots X_{n - 1}^r,X_n^t)$ to represent the path information, where $X_i^t$ is a node, and $X_i^r$ is the relation. $X_1^t$ is the starting node, $X_3^t$ is the related nodes obtained by biased random walk.

### GRU-Cooperation-Attention-Network

**Fig. 1:**
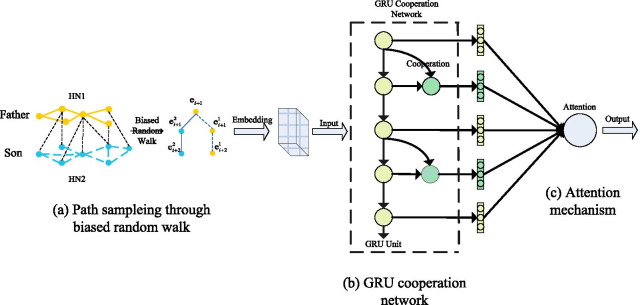
The architecture of GCAN

As shown in Fig. 1, we use the RNN(Recurrent Neural Network)-based model to predict drugs, because RNN-based model have stable and excellent performance in sequence prediction. We use the path information of the node as the input of RNN. At current time, the output of RNN is:

1 $$\begin{aligned} {h_t} = \tanh ({W_h}{h_{t - 1}} + b) \end{aligned}$$

where $W_h$ is the weight, $h_{t - 1}$ is the hidden state of previous node and *b* is a bias term. We use the GRU network to model this problem, GRU is a variant network of RNN by adding a gating mechanism to the network. GRU can more effectively optimize the spread of hidden information and mine the deep potential information of sequence. Considering that the GRU network will process the components of the sequence information indiscriminately, which means that the GRU network will treat the nodes and relationships in the relation-entity chain as an element. In this case, how to fully mine data information is a key issue. Therefore, we use the cooperation mechanism, which allows the input of current node $X_t$ to participate in the prediction, and at the same time, it can directly participate in the prediction by adjusting the weights, which can more fully mine the data and obtain the information. Given the hidden state of the previous node $h_{t - 1}$ and the input $X_t$, we can obtain the hidden layer $h_t$ at time *t* by the following formula.

2 $$\begin{aligned} {h_t} = \tanh ({W_h}{h_{t - 1}} + {W_x}{X_{\mathrm{t}}}{{ + }} b) \end{aligned}$$

We predict the drugs for the treatment of diseases based on the information of the existing node’s association chains. Not all the node information obtained during the biased random walk has a key effect on the predictive ability of the model. To optimize the weight of different nodes in the model prediction process we use attention mechanism to process the output from the GRU cooperation network. Our method could perform weighting operations for each predicted result. The weight vector formula at *t* time is:

3 $$\begin{aligned} {\alpha _{\mathrm{ti}}}{ =\mathrm { h}}_t^{\mathrm{T}}{W_\alpha }{h_{\mathrm{{i}}}} \end{aligned}$$

The node vector formula is:

4 $$\begin{aligned} {\mathrm{c}_{\mathrm{t}}}{{ = }}\sum \limits _{i = 1}^{t - 1} {{a_{ti}}{h_i}} . \end{aligned}$$

Deep learning networks can automatically fit the values of weights. And we can use the softmax function to obtain the attention weight vector, then the formula becomes:

5 $$\begin{aligned} {\alpha _{\mathrm{t}}}{{ = }}S\mathrm{oft}\max ([{\alpha _{t1}},{\alpha _{t2}}, \ldots {\alpha _{t(t - 1)}}]) \end{aligned}$$

### Biased random walk

**Fig. 2:**
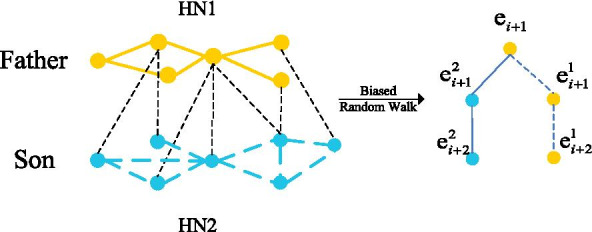
Biased random walk: Father nodes are more inclined to collect relationship chain information from the Son node

As shown in Fig. 2, to obtain more correlated path information, we choose to sample the relationship path deeper and more biased direction toward the father and son nodes. To this end, we use two HN data sets $HN_1=(h_1,r_1,t_1)$, $HN_2=(h_2,r_2,t_2)$to provide enough space for walking. To allow the random walking mechanism to find the father node, we set up a relationship subset to bridge two HN data sets through the mapping with the father–son relationship node: $S \subset HN_1 \times HN_2$ The walking direction of the conventional random walking mechanism [25] follows the following probability distribution:

6 $$\begin{aligned} \Pr ({e_{i + 1}}|{e_i}) = \left\{ \begin{array}{l} \frac{{{\pi _{{\mathrm{e}_i} \rightarrow }}_{{e_{i + 1}}}}}{N}\exists \qquad r \in R:({e_i},r,{e_{i + 1}}) \in G\\ 0 \qquad \qquad \qquad \mathrm{otherwise} \end{array} \right\} \end{aligned}$$

where $e_i$ is the first node, $e_{i+1}$ is the collected node, *Pr*() is the function of probability distribution. The conventional random walk mechanism is only subject to depth-first search or breadth-first search, which often only use one-sided path information, and it is impossible to obtain the information of neighbor nodes comprehensively. Considering the importance of the father and son nodes of rare diseases, we employ biased random walk, which combine with breadth-first search and depth-first search, to smoothly control the nodes. When we search for the neighbor nodes of $e_{i}$, the candidate nodes include $e^{1}_{i+1}$ in the same network, and the father node $e^{2}_{i+1}$ in another HN. Because we are more inclined to find father–son nodes and the deeper nodes, the final searched node is $e^{2}_{i+1}$. The biased walk mechanism obeys the following probability distribution:

7 $$\begin{aligned} {\mathop {\mathrm{P}}\nolimits } ({e_{i + 1}}|{e_i}) = \left\{ \begin{array}{l} \partial {{ \,\qquad \qquad (}}{\mathrm{e}_i},{e^2}_{i + 1})\\ 1 - \partial {{ \,\, \qquad (}}{\mathrm{e}_i},{e^1}_{i + 1}) \end{array} \right\} \end{aligned}$$

where $e_{i}$ represents the target node, $e^{2}_{i+1}$ and $e^{1}_{i+1}$ indicates father node and son node, respectively.

8 $$\begin{aligned} L\,\,=-\sum _{t=1}^{T-1}{\left\{ \log \sigma \left( h_{t}^{\prime }y_t \right) +\sum _{j=1}^k{\left\{ E_{{\bar{y}}_j{\tilde{q}}\left( {\bar{y}} \right) }\left[ \log \sigma \left( -h_{t}^{\prime }y_t \right) \right] \right\} } \right\} } \end{aligned}$$

where $y_{t}$ represents the predicted target at time *t*, $\sigma (\cdot )$ indicates sigmoid function, *k* is the number of negative samples, $q_{(yi)}$ is the sample obtained from the noise probability distribution, $y_{i}$ is the occurrence frequency in the data.

## Results

### Experimental setting

To match as much data similar to rare diseases as possible, we extracted data of 24 rare diseases from the data provided by Peking Union Medical College Hospital, and found father–son relationships from the existing HNs. We also used drug or gene as keywords to look for relevant disease data on DrugBank to add to the data. Finally, we got 7000 tuples of related data. The data were divided into training set and test set in a ratio of 2:1. The hidden layer size of the neural network is 256, the number of layers is 2, the size of batch is 512 and learning rate is 0.003.

The experiments were implemented on a computer with an Intel(R) Core(TM) i7-8700CPU processor, and an NVIDIA GeForce GTX Titan Xp GPU card with scalable link interface (SLI).

We choose *Hits*@10 and mean reciprocal rank (*MRR*) as the evaluation metrics. *Hits*@10 indicates the proportion of the results in the test set among the top-10 prediction results. *MRR* only considers the top real matched ratio in the prediction results.

### Comparison with state-of-the-arts

**Table 1:** Drug repositioning results of GCAN and many traditional HN embedding methods

Methods	Hits@10	MRR
GCN-AlignE [38]	0.067	0.042
TransR [31]	0.124	0.143
MtransE [35]	0.204	0.187
BootEA [36]	0.272	0.199
RSN [39]	0.403	0.205
GCAN	0.454	0.231

We compared GCAN with many traditional HN embedding methods such as GCN-Align [38], TransR [31], MtransE [35], BootEA [36] and RSN [39]. The drug repositioning results are shown in Table 1. GCN-Align is a convolutional computational model on graph nodes that does not exploit the semantic information of the nodes. TransR and MtransE both belong to path-level models and have good interpretability and good predictive ability. BootEA and RSN are improved on the basis of the previous models to increase accuracy. Table 1 shows that the performance of GCN-Align is poor, because it does not consider the importance of the relationship in model prediction, and the convolution-based method is not as reliable as the path-level embedding method. Both TransR and MtransE are improved methods based on TransE. Although they belong to path-level models, they also ignore the importance of relational information in the prediction process. Comparing with some recent models, they are relatively simple and cannot fully mine the hidden information from data, so the accuracy of the model is still low. Therefore, we tried some recent models such as BootEA and RSN. The predictive ability of these models has been significantly improved compared with the previous models because they can mine data information more deeply. RSN model adds the relational information to participate in the prediction, so it performs better than the previous model. What’s more, we improved GRU-network for RSN. Table 1 shows that GCAN has the best performance under Hits@10 and MRR. The accuracy of the RSN model has increased by 5.1%, which has valuable reference for drug repositioning of rare diseases. To further explore the influence of the data link in the model, we use biased random walk at different depths and employ cooperation attention mechanism to explore the hidden information from data. The experimental results in Table 1 show that the above improvements can significantly enhance the performance of GCAN model for drug repositioning.

### Ablation study

**Table 2:** Drug repositioning results using different modules

Methods	Hits@10	MRR
RSN	0.403	0.205
GCAN-normal-random-walk	0.425	0.213
GRU-Cooperation-Network	0.443	0.226
GCAN	0.454	0.231

To illustrate the effectiveness of each proposed module, we conduct a detailed analysis next. The results using different modules are shown in Table 2. RSN is the baseline model using RNN network. When we use GRU network instead of RNN network for modeling, it can achieve a better performance, because GRU network can obtain the long-term memory information for prediction. On this basis, we add a cooperation mechanism to enhance the ability to minimize hidden information. The result shows that it can significantly improve the ability of our model. Among them, the collection of data information is particularly important in the whole work, so we tried to use an ordinary random walk to collect path information, and the result shows that it is quite different from the accuracy of our biased random walk. Moreover, to improve the accuracy of the model, we use an attention mechanism to calculate the output of the GRU network and assigns weight to the results. Experimental results prove that the attention mechanism enhances the predictive ability of the model. The above experimental results prove that each of our works are essential for drug repositioning.

### The effect of random walk length

**Fig. 3:**
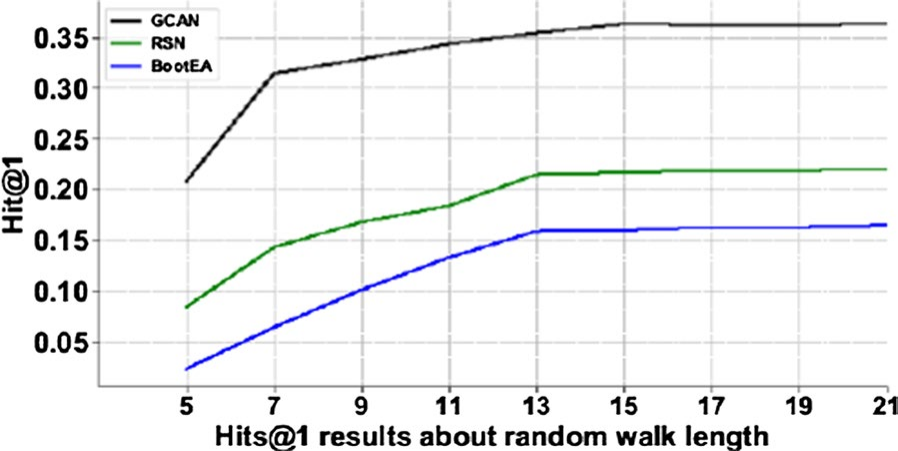
Under the hit@1 evaluation index, the influence of walking depth from 5 hops to 21 hops

To explore the most efficient walking depth, we explored *Hits*@10 from 5 to 21 hops as shown in Fig. 3. The accuracy of GCAN increases faster before 15 hops because longer path is useful for the expansion of the information, which can more fully explore the links between data. But after 15 hops the accuracy of the model grows very slowly and the time of calculation increases significantly. The experimental results of RSN and BootEA models also demonstrate the similar rule. Therefore, we finally chose the depth of 15 hops considering the calculation cost.

### Case study

**Fig. 4:**
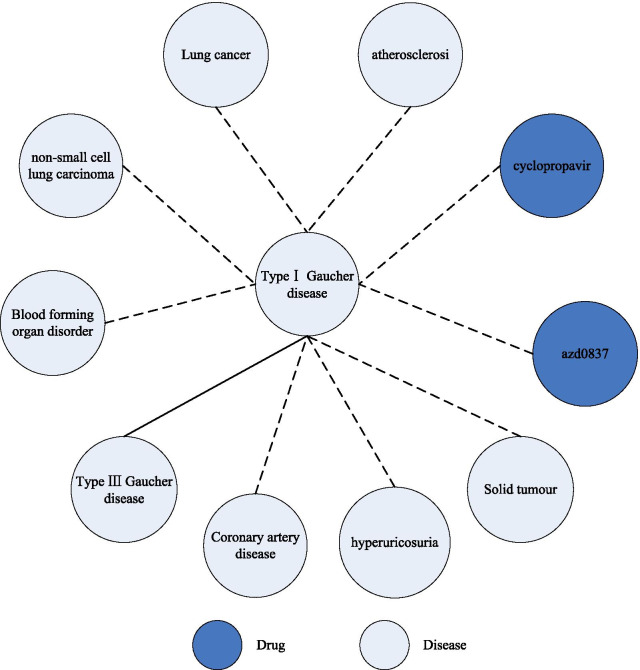
The pathway information and prediction results for drug repositioning of Gaucher disease

We show the process of drug prediction by GCAN model using Gaucher disease as an example. The pathway information and prediction results for drug repositioning of Gaucher disease are shown in Fig. 4. In Fig. 4, the solid line indicates the existing relationships in HNs, and the dashed line indicates the relationships predicted by GCAN model.

Gaucher disease includes type I Gaucher disease and type III Gaucher disease, which are father–son nodes in the network. In the embedding representation phase, we connect nodes with two hop distances in a hierarchical relationship in two HNs and use a biased random walk mechanism to collect information from the father and child nodes. When using type III Gaucher disease as the starting node on two HNs with biased random walking, the adjacent type I Gaucher disease nodes and their therapeutic drugs will be collected first to perform prediction. As shown in Fig. 4, two drugs for type I Gaucher disease predicted by GCAN are proved to be effective. The results proved that GCAN maintains the predictive capability for long-range nodes while enhancing the weight of short-range node information in the network layer.

## Discussions

In this paper, we use GCAN to investigate drug repositioning for rare diseases. GCAN captures the path information of disease nodes smoothly using a biased random wandering mechanism, and place more emphasis on feature capture of father–son node information. The experimental results shows that GCAN significantly outperformed the state-of-the-art HN embedding methods. Because GCAN addresses the problem of sparse rare disease data and makes full use of father–son information to augment the data size as well as the connectivity between data. In the experiments, we expand the scale of the data by using two different HNs, one HN contains the father nodes of the disease and the other one contains the son nodes. The father–son nodes are used as a bridge to connect the two HNs and control the direction of node path collection. Using two HNs enrichs the path information of nodes in the path acquisition process. In addition, the GRU cooperative attention mechanism optimizes the weight distribution of path information in the propagation process and focuses more on learning the feature information of nodes that have father–son relationship with the rare disease nodes, which enhances the prediction ability of the model for rare diseases.

The experimental results also show that the performance of GCAN is still limited by insufficient scale and low quality of the data. The existing data need to be improved in both scale and quality. However, it is difficult to obtain a large scale of data related to rare diseases. Therefore, the future work for drug repositioning in rare diseases is to collect more valuable data and to make better use of the current limited data

## Conclusion

In this paper, we proposed a HN embedding model called GCAN to perform drug repositioning for rare diseases. GCAN enhances the mining of hidden information about rare diseases through biased random walk mechanism, GRU-cooperation mechanism and attention mechanism. The drug repositioning experiment shows that GCAN significantly outperforms the existing HN embedding methods. The performance of GCAN model is still limited by the scale and quality of the data. In the future we will employ additional data such as protein structure information in combination with current data for drug repositioning of rare diseases.

## Data Availability

The datasets belong to a third-party and the authors do not have permission to share the data.

## Abbreviations

GRU: Gate Recurrent Unit
GCAN: GRU Cooperation Attention Network
HN: Heterogeneous network
CNN: Convolutional neural network
RSN: Recurrent skipping network
RNN: Recurrent neural network
MRR: Mean reciprocal rank
